# Micro-CT-derived ventilation biomarkers for the longitudinal assessment of pathology and response to therapy in a mouse model of lung fibrosis

**DOI:** 10.1038/s41598-023-30402-8

**Published:** 2023-03-17

**Authors:** Francesca Pennati, Ludovica Leo, Erica Ferrini, Nicola Sverzellati, Davide Bernardi, Franco Fabio Stellari, Andrea Aliverti

**Affiliations:** 1 Dipartimento di Elettronica, Informazione e Bioingegneria, Politecnico di Milano, Milano, Italy; 2 Department of Medicine and Surgery, University of Parma, Parma, Italy; 3 Department of Veterinary Science, University of Parma, Parma, Italy; 4 Pharmacology and Toxicology Department Corporate Pre-Clinical R&D, Chiesi Farmaceutici S.P.A., Largo Belloli 11/A 43122, Parma, Italy

**Keywords:** Preclinical research, Image processing, Diagnostic markers, Respiratory tract diseases

## Abstract

Experimental in-vivo animal models are key tools to investigate the pathogenesis of lung disease and to discover new therapeutics. Histopathological and biochemical investigations of explanted lung tissue are currently considered the gold standard, but they provide space-localized information and are not amenable to longitudinal studies in individual animals. Here, we present an imaging procedure that uses micro-CT to extract morpho-functional indicators of lung pathology in a murine model of lung fibrosis. We quantified the decrease of lung ventilation and measured the antifibrotic effect of Nintedanib. A robust structure-function relationship was revealed by cumulative data correlating micro-CT with histomorphometric endpoints. The results highlight the potential of in-vivo micro-CT biomarkers as novel tools to monitor the progression of inflammatory and fibrotic lung disease and to shed light on the mechanism of action of candidate drugs. Our platform is also expected to streamline translation from preclinical studies to human patients.

## Introduction

Experimental in-vivo animal models^1,2^ have been instrumental to achieve detailed insight into the pathogenesis of lung fibrosis and to discover novel therapeutics. Among them, the bleomycin (BLM)-induced pulmonary fibrosis mouse model is the most widely used and internationally recognized^1^. Currently, histopathological and biochemical analysis of explanted lung tissue represent the gold standard to assess fibrosis development and progression^3,4^. However, ex-vivo analyses provide a space-localized and static information and are limited to one measurement at a given time-point, precluding longitudinal studies in individual animals. Moreover, the results of standard analyses strongly differ from clinical endpoints such as pulmonary function tests and high resolution-computed tomography (HRCT), thus underlining the need for more clinically relevant parameters. Although HRCT is an invaluable tool for the assessment of several lung disorders in clinical practice, it is mainly used as a diagnostic tool in pulmonary fibrosis^5–7^. This is mainly due to its challenging interpretation, which heavily relies on radiologist’s expertise, lack of objectivity and poor quantification. For these reasons, HRCT is presently not fully integrated into clinical trials to assess fibrosis progression and to evaluate the efficacy of pharmacological treatments. Despite these current limitations, quantitative analysis of CT images has the potential to provide important physiopathological clues and clinically relevant readouts.

In preclinical settings, micro-computed tomography (micro-CT) is being increasingly used to investigate lung disease in small animals and has already demonstrated its potential as a suitable tool for longitudinal studies, capable of providing qualitative and quantitative data to track disease progression as well as response to therapy. Although different quantitative measurements have been proposed for different lung disease models, such as the amount of air entrapped within the lungs^8,9^, hyperdense lung volume^10^, total lung volume^9,11^ and mean lung density^9,11,12^, they all rely on single end-expiratory phase, thus precluding any further assessment of lung function. Other approaches based on the analysis of the distribution of tissue density have been proposed to discriminate between aeration compartments^12,13^ and used for dynamic evaluations in free-breathing animals^14^.

Recently, measurements of regional differences in intensity between images acquired at different lung volumes by non-contrast CT has been proposed as a tool for imaging ventilation in human subjects. Ventilation imaging has been investigated in different lung diseases such as non-small cell lung cancer^15,16^, COPD^17,18^, asthma^19^ and post-lung transplantation^20,21^, demonstrating its potential to assist diagnosis as well as to inform on lung disease progression and to follow the outcome of intervention therapy. In preclinical studies, instead, in vivo lung function measurements by flexiVent are mainly used to quantify the extent of ventilation impairment, providing information related to the respiratory system mechanics (i.e. compliance and resistance) through the analysis of pressure and volume signals^22^. However, this represents a terminal technology applied to intubated and mechanically ventilated animals, and although repeated measurements are possible, the resulting data could be affected by specific technical aspects (and variables) inherent to the procedure^23–25^.

**Figure 1:**
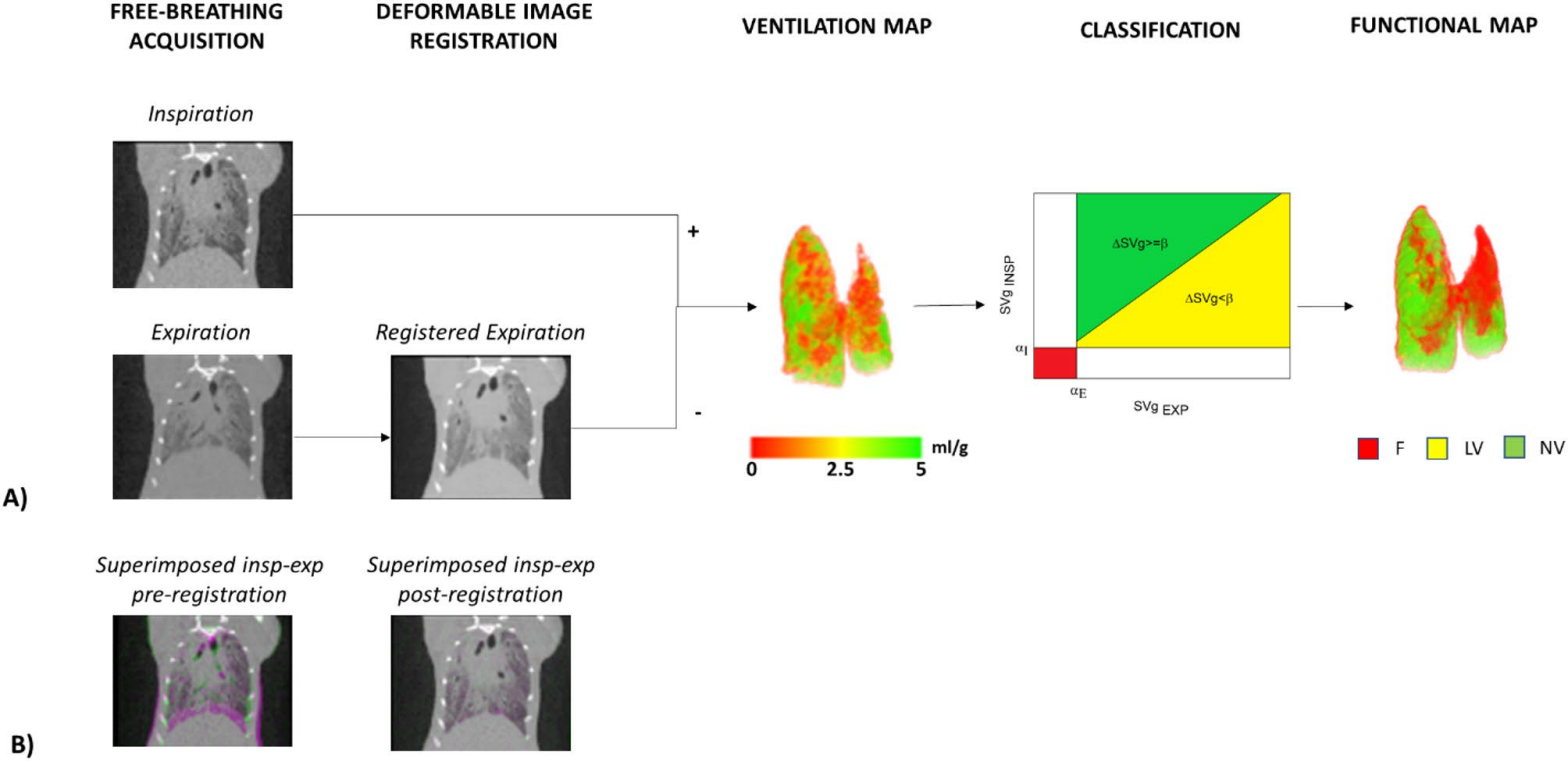
(**A**) The image processing algorithm. The images are acquired in free-breathing and reconstructed at peak inspiratory and peak expiratory states. Deformable image registration is applied to match corresponding voxels in the expiratory and the inspiratory images. After converting the images in specific gas volume, the ventilation map is obtained by subtracting the deformed expiratory from the inspiratory image. Based on DSVg and inspiratory and expiratory SVg values, each voxel of the lung is classified as fibrosis (red), low ventilation (yellow) and normal ventilation (green). (**B**) Superimposed inspiratory and expiratory images before and after registration, with non-matched regions pre-registration (right) coloured in pink(near the diaphragm).

The goal of the present study was to derive ventilation parameters from micro-CT scans corresponding to the end-inspiration and end-expiration phases in healthy and fibrotic BLM-treated mice (see Fig. 1 for an outline of the overall procedure, which is explained in detail in“Materials and Methods”). More specifically, our aim was to extract functional respiratory parameters capable of longitudinally and quantitatively profiling fibrosis progression and response to the human use-approved antifibrotic drug nintedanib (NTD) in the whole lung and in the upper and lower lung regions, and to compare the data thus acquired with histomorphological endpointdata.

As suggested by the results presented in this work, the acquisition of quantitative and more comprehensive (whole-lung and region-specific) data by micro-CT can provide highly informative readouts with a high potential for translation into clinical practice.

## Results

**Figure 2:**
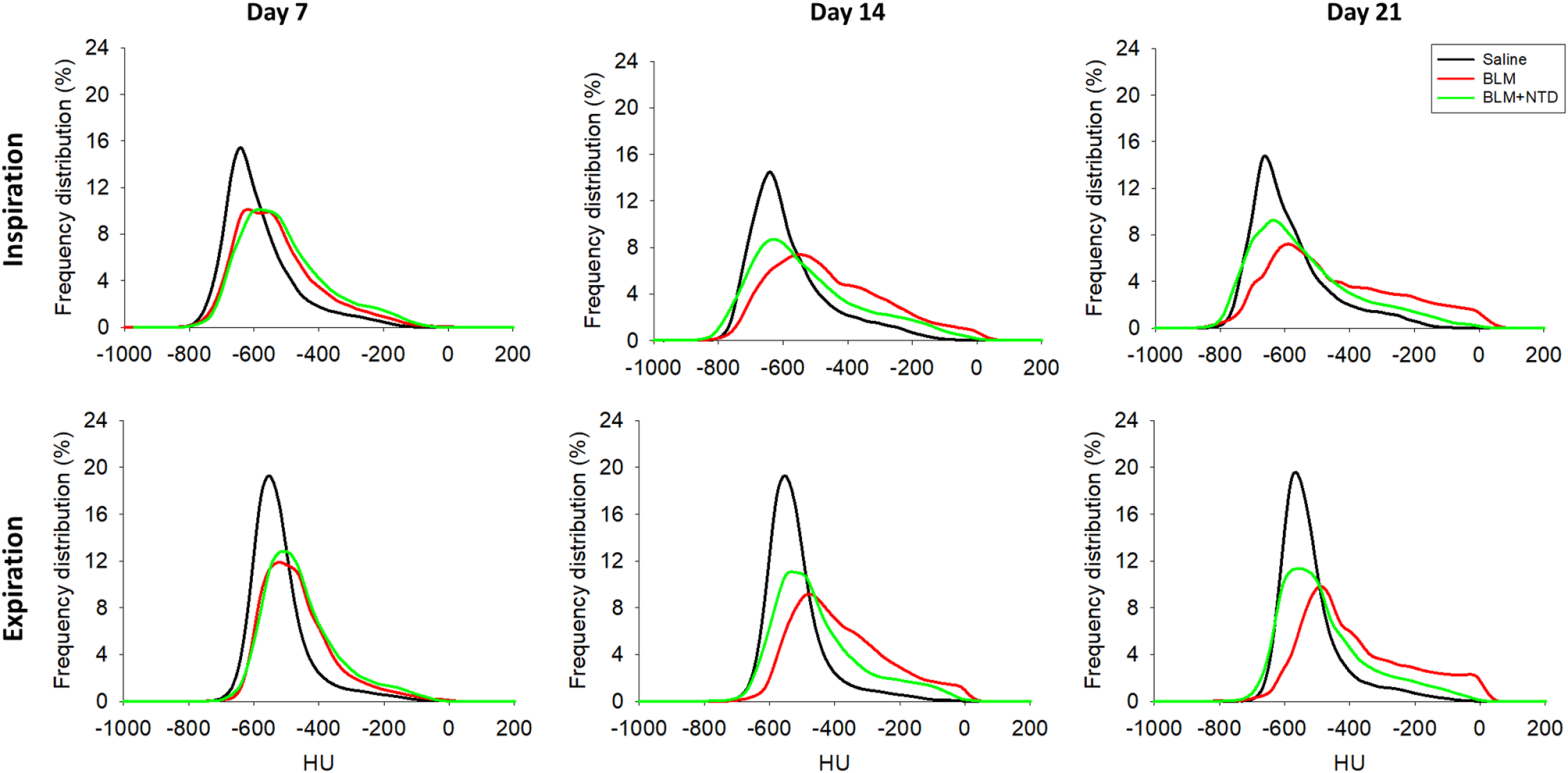
Median density histograms of saline (black), BLM (red) and BLM + NTD (green) groups in inspiration (top row) and expiration (bottom row) at all timepoints. The histograms showed a progressive shift of the peak to the right starting from day 7 and a thickening of the right-side tail from day 14 for both the BLM and the Nintedanib-treated groups compared to the saline. At day 21, these changes progressed only in the BLM group.

We imaged individual mice (eight BLM- and BLM + NTD-treated animals plus five saline controls) longitudinally at 7, 14 and 21 days. The whole-lung median density distributions for the three groups at the three time-points are reported in Fig. 2 both in inspiration (*top row*) and expiration (*bottom row*). At day seven, before start of the nintedanib treatment, the BLM and the BLM + NTD histograms appeared to be similar, with a right-shifted, smaller size peak compared to the saline control, indicating an overall increase in CT attenuation values. Despite the limited tidal volume expected for free-breathing mice, micro-CT was able to detect the small air lung variation relative to saline controls, which resulted in a left-shift of all histograms in the inspiration phase. At day 14 and 21, the saline group histograms remained unchanged both in inspiration and expiration. The BLM + NTD histograms progressively shifted toward the saline values, whereas a progressive shift of the peak to the right, accompanied by a thickening of the right-side tail, was observed in BLM-treated animals.

**Figure 3:**
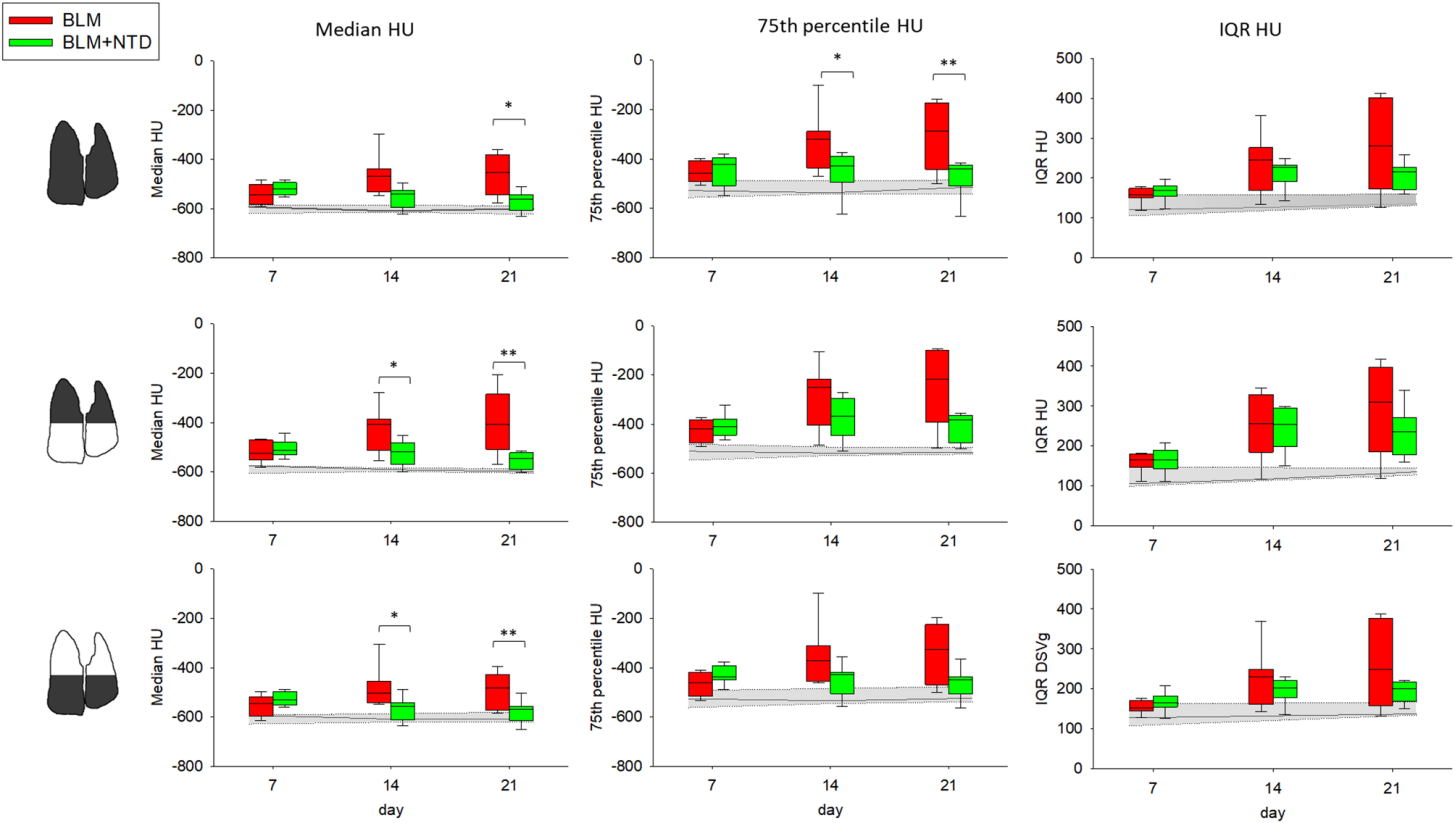
Median (first column), 75th percentile (second column) and IQR (third column) of HU computed in inspiration, in the BLM (red) and BLM + NTD (green) group. The analysis was performed separately for the overall lung (top row), upper lung regions (middle row) and lower lung regions (bottom row). Gray area represents the saline values and is delimited by the 25th and the 75th percentile.

Lung density biomarkers confirmed these observations (Fig. 3). At the whole-lung level (*upper row*), BLM-treated animals featured a higher median (*first column*), 75th percentile (*second column*) and IQR (*third column*) density compared to the saline controls at all time-points, and significantly higher median and 75th percentile at day 21 compared to NTD-treated animals. By separately considering the upper and the lower lung regions (Fig. 3, *middle* and *bottom* rows, respectively), significant median density differences between BLM and NTD treatment groups became apparent in both regions, both at day 14 and 21.

**Figure 4:**
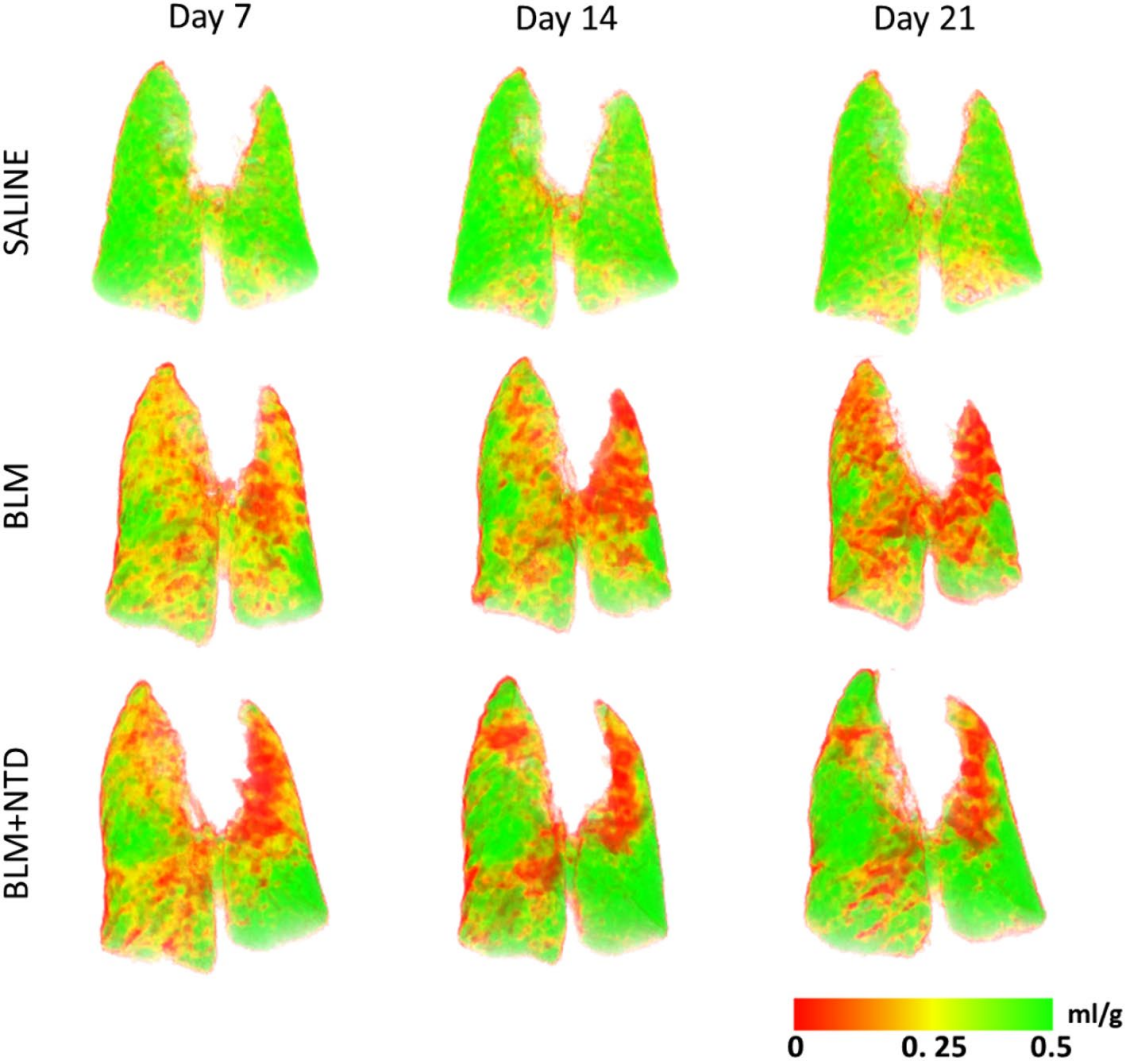
Ventilation maps in a saline (top) and a representative BLM (middle) and BLM + NTD (bottom) animal at all timepoints. Color scale indicates the SVg difference between the inspiratory and the expiratory lung images (ml of air to grams of tissue). In the saline the ventilation map is homogeneously coloured in green (about 0.4 ml/g). In the BLM animal, at day 7, an overall decrease of ventilation was present in the overall lungs (about 0.2 ml/g), which progressed in the following timepoints, with apical regions at day 21 not ventilated. In the BLM + NTD animal, ventilation decreased similarly to the BLM at day 7, but was partially recovered at day 14, remaining stable at day 21.

Inspiratory-expiratory ventilation maps extracted from micro-CT images were then used to unveil lung functionality of regions with increased tissue density (see the representative images shown in Fig. 4). Saline controls (*first row*) were characterized by a ventilation map indicative of normal ventilation (homogeneously coloured in *green*) with a DSVg value higher than 0.4 ml/g at all time-points. In the BLM (*second row*) and the BLM + NTD (*bottom row*) treatment groups, ventilation maps at day 7 were mainly coloured in *yellow-orange* with a DSVg around 1 ml/g, which is indicative of an overall decreased ventilation likely associated to the acute phase of the inflammatory process. At the subsequent time-points, BLM-treated animals featured a progressive worsening of ventilation, both in the left and the right lung, with large fibrotic (*red-coloured*) regions, corresponding to DSVg values below 1 ml/g. In contrast, a partial recovery of ventilation, with small fibrotic (*red*) regions only visible at the apexes of the lungs, was observed in BLM + NTD-treated animals on day 14 and remained stable till day 21.

**Figure 5:**
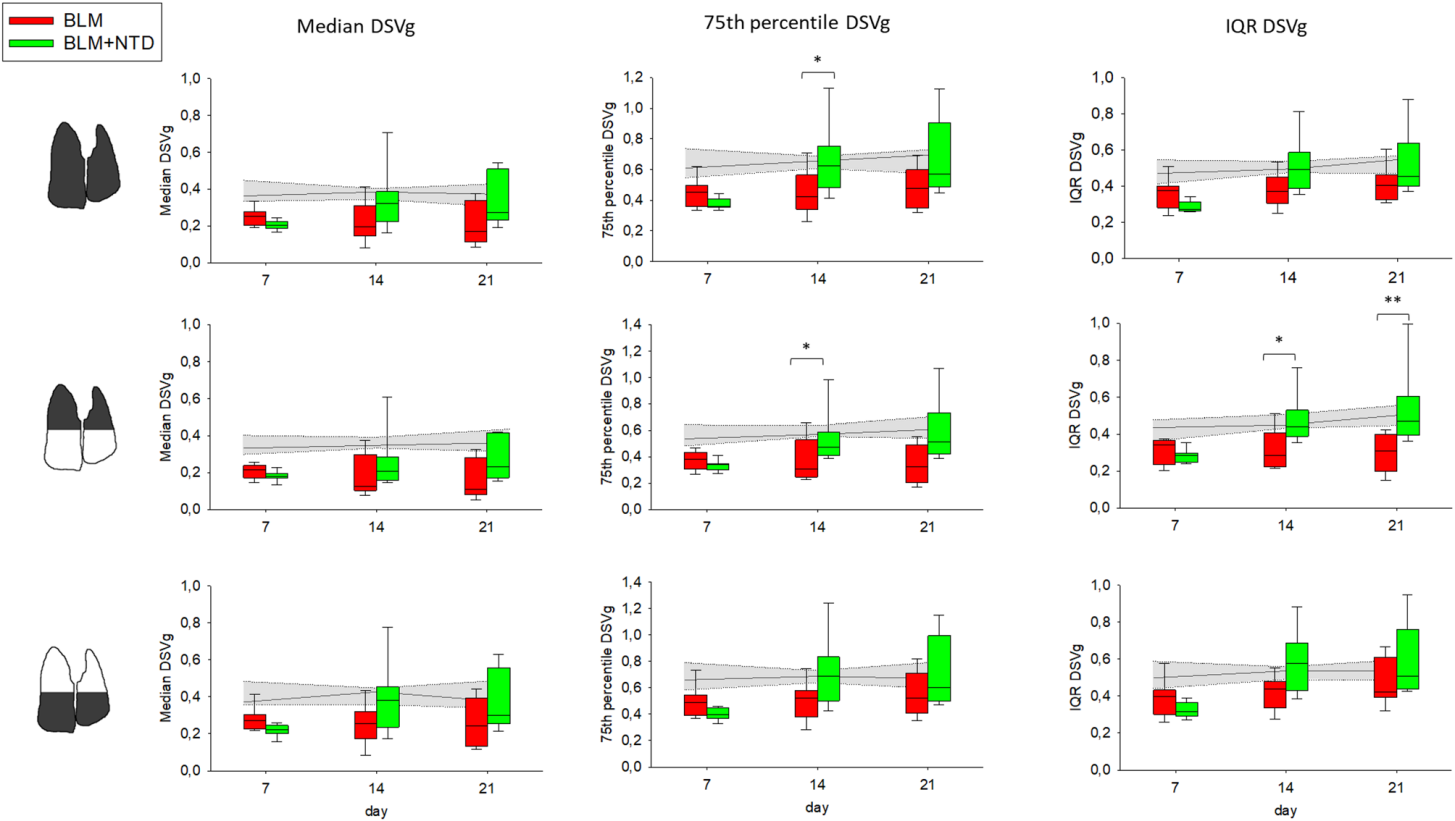
Median (first column), 75th percentile (second column) and IQR (third column) of DSVg, in the BLM (red) and BLM + NTD (green) group. The analysis was performed separately for the overall lung (top row), upper lung regions (middle row) and lower lung regions (bottom row). Gray area represents the saline values and is delimited by the 25th and the 75th percentile.

The longitudinal trend of the ventilation biomarkers is shown in Fig. 5. As expected, the BLM group had lower ventilation for all metrics and at all time-points compared to the saline controls. In contrast, NTD-treated animals had lower ventilation compared to the saline group only at day 7, and ventilation was restored to nearly normal average levels at the subsequent time-points, albeit with a marked intra-group variability. As revealed by 75th percentile and IQR values, at day 14 and 21, NTD-treated animals had higher ventilation in the upper lung regions compared to the BLM group.

**Figure 6:**
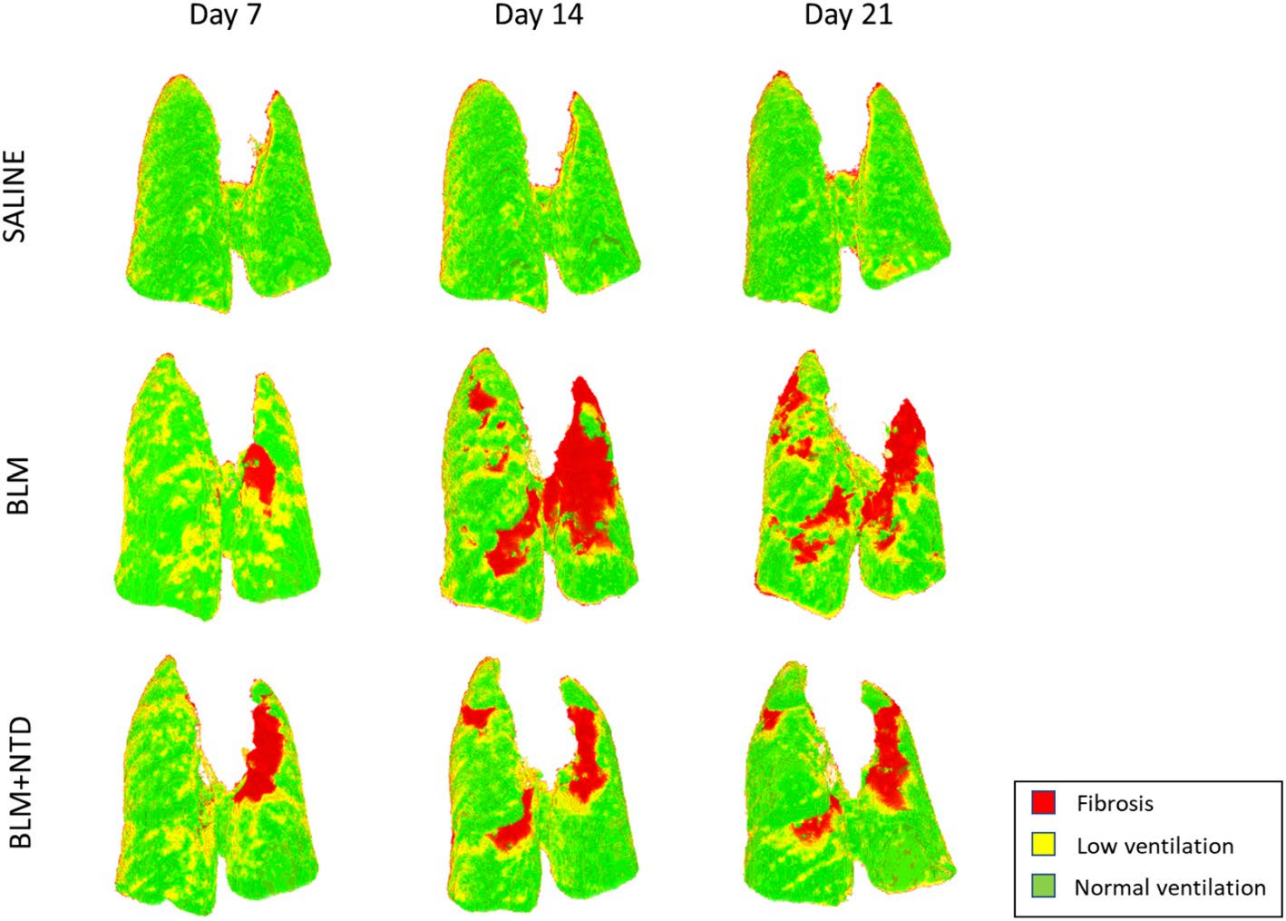
Functional maps in a saline (top) and a representative BLM (middle) and BLM + NTD (bottom) animal at all timepoints. In the saline, the lungs are normally ventilated. In the BLM animal, at day 7, On the functional map, only a small region of the left lung was fibrotic. At day 14, a large fibrotic region in the upper left lung was present, which widened at day 21, also affecting the right lung. In the BLM + NTD animal, a small fibrotic region was present on day 7 in the left lung, which remained stable in the following timepoints.

In order to differentiate between decreased ventilation due to a reduced density variation or to fibrosis formation, the ventilation map shown in Fig. 4 were converted in functional maps and reported in Fig. 6. As expected, at all time-points, the saline control was classified as normally ventilated. At day 7, even if both the BLM and the BLM + NTD showed an overall decrease of ventilation, only a small amount of fibrosis (*red*) in the apical regions was observed in both the cases. At later time-points, the fibrotic region progressively widened in the BLM-treated animal, also affecting the right lung, whereas it remained stable in the BLM + NTD-treated mouse.

**Figure 7:**
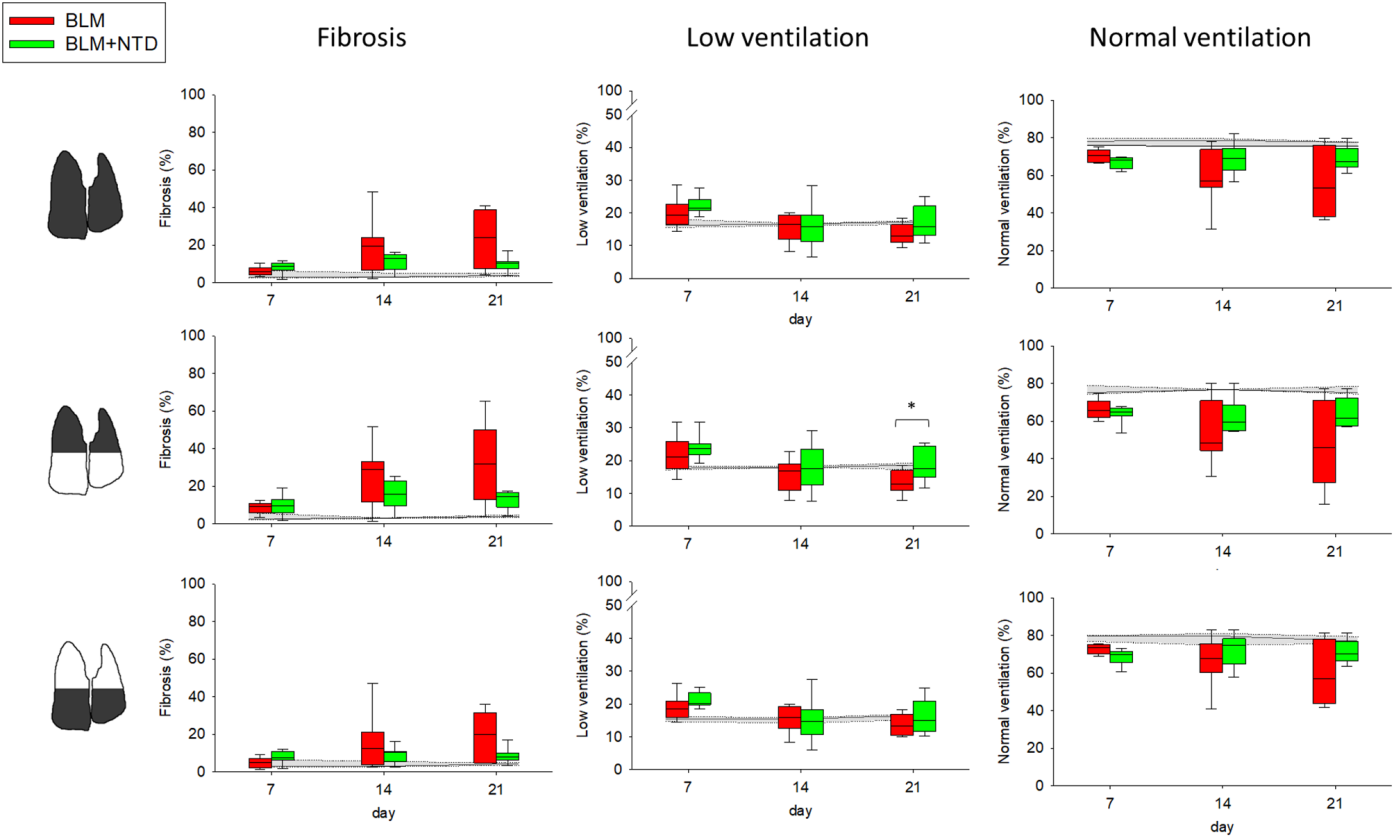
Percent extension of fibrosis (first column), low ventilation (second column) and normal ventilation (third column) in the BLM (red) and BLM + NTD (green) group. The analysis was performed separately for the overall lung (top row), upper lung regions (middle row) and lower lung regions (bottom row). Gray area represents the saline values and is delimited by the 25th and the 75th percentile.

A cumulative, longitudinal quantification of the functional regions is reported in Fig. 7. Fibrosis (*first column*) was significantly more pronounced in the BLM-treated compared to the saline control group at day 14 and 21 (*p* < 0.001) with an intra-group variability progressively increasing from day 7 to day 21. Fibrotic areas in the BLM + NTD-treated animals remained quite stable from day 7 to day 21 with no significant differences with respect to the saline and the BLM groups. Low ventilation (*second column*) was significantly higher in the BLM and the BLM + NTD groups compared to the saline controls only at day 7 (*p* < 0.001, both in the upper and the lower regions). Normal ventilation was lower in both treatment groups compared to saline at all time-points but only in the upper regions. In the lower regions, Nintedanib-treated animals recovered the ventilation values of the saline controls, thus suggesting compensatory regional effects.

**Figure 8:**
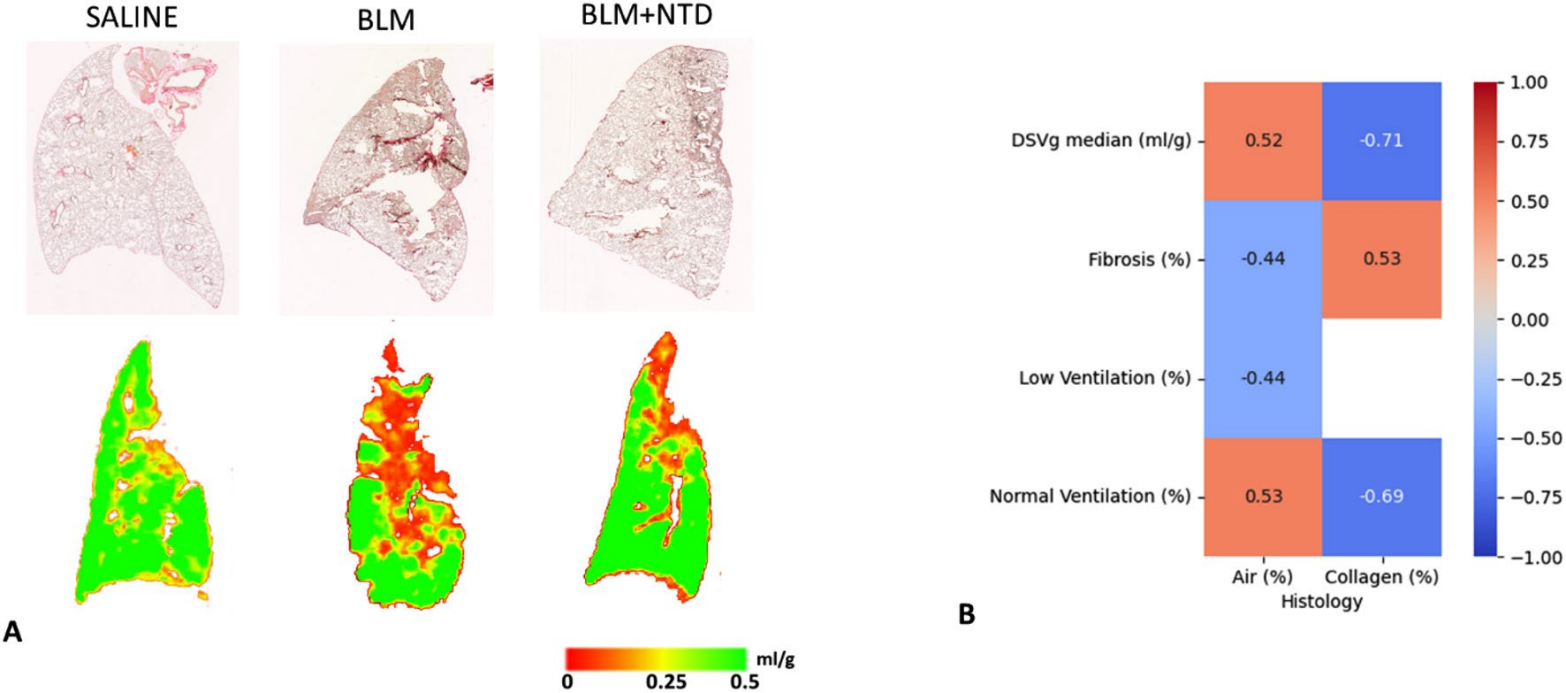
On the left, histological sections were reported with the corresponding ventilation maps, at day 21, showing that regions with moderate and severe lesions are characterized by low/no ventilation. Colour scale of ventilation images indicates the SVg difference between the inspiratory and the expiratory images (ml of air per gram of tissue). On the right, Spearman matrix calculated between ventilation parameters and histological measures. The colour bar evidenced high positive correlations in red and high negative trends in blue. The correlation coefficient (r) is reported in each box, when significant.

The correlation between ventilation and histomorphometric parameters derived from analysis of three equally sized regions of the left lobe is reported in Fig. 8. Ventilation and histomorphometric measures are reported in supplemental Table 1. Histological sections with the corresponding ventilation maps at day 21 are shown on the left, where it can be seen that regions with moderate or severe lesions are characterized by low or no ventilation, respectively. Median ΔSVg correlated with both the collagen content (r = − 0.71, *p* < 0.0001) and the alveolar air (r = 0.52, *p* = 0.0007, Fig. 8B). Collagen content negatively correlated with the normally ventilated percentage (r = − 0.69, *p* < 0.0001), while it positively correlated with the fibrotic areas percentage (r = 0.53, *p* = 0.0001). Alveolar air positively correlated with the normally ventilated percentage (r = 0.53, *p* < 0.001) and negatively with low-ventilated (r = − 0.44, *p* = 0.006) and fibrosis percentage (r = − 0.44, *p* = 0.006).

## Discussion

Micro-CT is a powerful tool for the in vivo investigation of animal models of lung disease, capable of providing longitudinal information on disease progression and the efficacy of novel therapeutics. In the present study, multivolume micro-CT was applied to a murine model of BLM-induced lung fibrosis and used to measure regional ventilation as inspiratory/expiratory lung density changes. Our findings demonstrate the ability of this approach to provide functionally relevant in vivo biomarkers allowing the longitudinal, region-specific quantification of lung (dys) function. Progression of BLM-induced alterations caused an overall, yet heterogeneous decrease of lung ventilation, due to the coexistence of low ventilation and fibrotic regions. Ventilation measurements also allowed to quantify the antifibrotic effect of Nintedanib, which resulted in a reduced accumulation of fibrotic lung tissue in treated mice. A good correlation between functionality and structural damage of the lungs was revealed by a parallel comparative analysis of micro-CT-based functional outcomes and histomorphometric endpoints. In vivo micro-CT biomarkers of regional ventilation may thus be used to quantitatively pinpoint the onset and progression of fibrotic inflammatory lung disease, thus allowing precise timing of antifibrotic treatments testing.

Micro-CT histogram data have previously been validated against gold standard histological parameters and proposed as alternative, quantitative biomarkers in specific lung disease models^8,13^. As expected, in the BLM group, histogram-based measurements revealed an increased distribution of pixels in more damaged areas of lung parenchyma, with tissue densities locally increasing from day 7 to day 21, reflecting both lung inflammation and fibrotic progression. In the Nintedanib-treated group, instead, a transient density increase at day 7, before treatment start, was followed by an appreciable recovery at subsequent time-points. The increasing interquartile range of the density distribution and the significant differences observed between the BLM and the BLM + NTD-treated groups, reflected the patchy distribution of the fibrotic lesions^26^, thus pointing to the superiority of micro-CT over histological and biochemical analyses with regard to the acquisition of spatially resolved information. Also, the observed intragroup variability indicated that the extent of parenchymal involvement is largely subject-specific, highlighting the advantage of in-vivo micro-CT as an in vivo tool to individually monitor disease onset and progression. Nevertheless, had micro-CT-derived measurements been limited to lung density, only tissue damage but no lung functionality information would have been extracted.

We addressed this limitation by implementing biomarkers of regional ventilation, based on inspiratory-expiratory changes in lung tissue density. In the BLM group, ventilation decreased starting from day 7. At the level of functional classes, we found a significantly higher percentage of low-ventilation at day 7, suggesting that this early decreased ventilation may be attributed to the acute inflammatory reaction caused by BLM^27^. At day 14 and 21, instead, a higher fibrosis percent, with a high intra-group variability, was apparent. Normal ventilation percentage markedly decreased at day 7 both in the apical and the lower regions, whereas at the subsequent time-points there was a raise in intra-group variability with significant lower values only in the apical regions, suggesting the appearance, in a subset of mice, of compensatory mechanisms aimed at maintaining the overall ventilation. In the BLM + NTD-treated group, ventilation was significantly lower compared to the saline controls at day 7, but at variance with BLM-only treatment, it improved at the subsequent time-points. Functional maps revealed that, as in the case of the BLM-only treatment, the decreased ventilation observed at day 7 was due to a large percentage of low-ventilation and a small percentage of fibrosis in the apical regions. At the subsequent time-points, a lower increase of fibrotic lung areas in the treated group was apparent. Altogether, these results indicate that a free-breathing imaging approach allows the non-invasive assessment of respiratory function, which represents a clear advantage over alternative methods based on mechanical ventilation, which may damage the trachea and the lungs^23^. We also found that compared to methods only relying on density analysis, the simultaneous evaluation of inspiratory and expiratory images allows to identify non-functional regions and to distinguish between truly fibrotic and low-ventilation (inflammatory) components, thus enabling a more detailed characterization of the alterations underlying the density changes occurring within the lung.

A possible weakness of the present study might be represented by the use of a semi-automatic segmentation method, which may prevent the application of the proposed micro-CT approach to larger datasets. We note, however, that neural networks-based algorithms for the automatic segmentation of lung tissue have recently been developed^28^, and their incorporation into the present work-flow would enable its conversion into a fully automated bioanalytical platform.

An important contribution of this work is the analysis of the correlation between ventilation maps and histological parameters, which highlighted a fairly robust structure–function relationship, with heightened collagen content and a reduced ventilation, due to an increase of both low-ventilation and fibrotic regions. Compared to histological analysis, micro-CT imaging enables 3D monitoring of individual animals throughout disease progression. We thus strongly support micro-CT as a diagnostic tool complementary to histology, which instead provides more accurate information at the cellular and molecular level. In future studies, the use of a more resolved time-course for histological analysis will allow a better characterization of the functional classes.

The thresholds we used to define the functional classes were chosen based on the distribution of density and ventilation in the healthy lung. Although each animal should be compared with its own baseline control to avoid variability, due to the low inter-subject variability observed in the saline group, the baseline values of the diseased lungs are comparable to that of the saline.^11^. Moreover, to take into account potential variations due to ageing-related processes ^11^, which may lead to an overestimate of pathological classes, we designed percentage thresholds instead of fixed values, which would allow to more easily adapt the thresholds to different experimental setting, such as older mice. It is also important to note, in this regard, that the same thresholds (or specifically adjusted variants thereof) could be used to translate micro-CT-based analysis to animal models with different parenchymal density. In summary, we have developed and validated an imaging procedure that uses micro-CT to obtain morphological and functional indicators of lung pathology and allows quantitative monitoring of disease progression in a murine model of lung fibrosis. The results of this exploratory study attest to the potential of this in-vivo imaging approach for drug screening. They also documented the advantages of micro-CT over other, most commonly histology-based techniques, as a tool to longitudinally portray the heterogeneity of pulmonary complications within different treatment groups and spatially within individual animals. The overall diagnostic performance of our micro-CT approach strongly supports its translation from preclinical studies to the clinical setting.

## Materials and methods

### Experimental design

#### Experimental animals

All studies were conducted in 8–9-week-old female inbred C57Bl/6 mice (purchased from Envigo, San Pietro al Natisone, Udine, Italy). Animals were housed five per cage upon arrival and acclimatized to the local vivarium conditions (room temperature: 20–24 °C; relative humidity: 40–70%; 12-h light–dark cycle; food and water ad libitum) for 7–10 days. All experiments were carried out in accordance with the intramural animal welfare practices for animal experimentation of Chiesi Farmaceutici and complied with the European Directive 2010/63 UE, Italian D.Lgs 26/2014 and the revised “Guide for the Care and Use of Laboratory Animals” (National Research Council Committee, US, 2011)^29^ and ARRIVE guidelines^30^. All animal procedures were conducted in an AAALAC (Association for Assessment and Accreditation for Laboratory Animal Care) certified facility at Chiesi Farmaceutici and were authorized by the Italian Ministry of Health with protocol number 841/2019-PR and by the internal AWB (Animal Welfare Body). All appropriate measures were taken to minimize pain or discomfort in the animals; the pain was evaluated daily through a Visual Analogue Scale (VAS) ranging from 0 to 10 by a designated veterinarian or trained technicians. Signs of dyspnoea, body weight loss ≥ 20% and VAS ≥ 6 were considered as humane endpoints.

### Lung fibrosis induction and pharmacological treatment

Pulmonary fibrosis was induced using bleomycin hydrochloride (Baxter, 1 mg/kg) diluted in 50 µL saline (0.9%) or vehicle via a double (day 0, 4) oropharyngeal administration (OA).

Briefly, animals were lightly anesthetized with 2.5% isoflurane delivered in induction chamber and positioned on the intubation stand. The tongue was pulled out with forceps, using a small laryngoscope and with a micropipette the liquid was placed onto the distal part of the oropharynx while the nose was gently closed, as previously described^31^.

All the mice were orally treated daily for 2 weeks (from 7 to 21 days) either with vehicle or Nintedanib at 60 mg/kg. All mice were weighed daily from the beginning of the trial to the end-point.

### Micro-CT acquisition protocol

Micro-computed tomography (micro-CT) lung imaging was performed longitudinally at day 7, 14 and 21 by Quantum GX Micro-CT (PerkinElmer, Inc. Waltham, MA). Each mouse was anesthetized using 2% isoflurane, which reduces respiratory rate and depth of ventilation, regularizing breathing^31^. After placing the animal in supine position on the scanner bed, the chest was aligned within the field of view and a respiratory region of interest is positioned over the diaphragm. The respiratory signal trace, the respiratory cycle lengths and the respiratory rate were monitored during the acquisition. Images were acquired with the following parameters: X-ray tube voltage 90 kV, X-ray tube current 88 µA and total scan time of 4 min. The retrospectively gated acquisition protocol was in ‘high speed’ mode, with projections collected in list-mode over a single continuous gantry rotation. Images were reconstructed using a filtered back-projection algorithm with a Ram-Lak filter into two 3D datasets, corresponding to the inspiratory and expiratory breathing phases, with 50 μm isotropic reconstructed voxel size. The reconstructions quality is reviewed on the transaxial, coronal and sagittal views to be sure not to have blurred images from movements due to the low level of anesthesia and, if necessary, the scan is repeated.

### Micro-CT image processing and analysis

Image processing and quantitative analysis were performed using the open-source National Library of Medicine Insight Segmentation and Registration Toolkit^32^. The image processing and quantitative analysis is outlined in Fig. 1A.

- Free-breathing acquisition. Images reconstructed at peak inspiratory and peak expiratory phases were converted from grey levels to Hounsfield Units (HU). Conversion was based on a linear transformation, in which 1000 HU and 0 HU were set as the density of water and air, respectively.
- Deformable image registration. To take into account the change of density between expiratory and inspiratory images, spatially equivalent pulmonary regions were matched by applying deformable image registration, which consists in finding the best spatial mapping match between spatially equivalent voxels in two images. The Demons algorithm^33^ with a four-level multiresolution strategy was applied. To make the registration more sensitive to structures than to overall intensity (which changes with lung volume), images were pre‐processed using a Laplacian filter^18^. The deformation field resulting from the registration algorithm was applied to deform and superimpose the expiratory onto the inspiratory image. Figure 1B shows the superimposed inspiratory and expiratory images before (left) and after (right) registration, with non-matched regions at the diaphragm level coloured in pink and only present in pre-registration. The accuracy of the registered images was validated both by reviewing each registration result for the correct alignment of visual anatomy and by calculating the target registration error (TRE) on anatomical paired landmarks. To calculate the TRE, we manually selected 10 pairs of landmarks between the inspiratory and the expiratory images, for each micro-CT dataset. Landmarks were placed from the diaphragm to the lung apexes, at the bifurcations of pulmonary vessels and airways. On average, TRE was 370 ± 377 µm before registration and 44 ± 34 µm after registration, with a level of accuracy comparable to the voxel resolution (50 µm).
- Ventilation map. After manual segmentation to separate lung parenchyma from the surrounding soft tissue, images were converted to specific gas volume (SVg)^34^ and subtracted on a voxel-by-voxel basis (ΔSVg = SVg_insp_—SVg_exp_)^18^, resulting in a ventilation map. For each voxel, ΔSVg represents the change in the amount of air relative to tissue mass between the inspiration and the expiration images. SVg was calculated as SVg = SV_tissueandgas_—SV_tissue,_ where SV is the specific volume (expressed in ml/g) and is calculated as the inverse of density (g/ml). The specific volume of tissue was assumed to be equal to 1/1.065 = 0.939 ml/g^35^ and the specific volume of the lung (tissue plus gas) was derived from CT images^34^: SV_tissueandgas_ (ml/g) = 1000/(HU + 1000).
- Classification and functional map. To differentiate between regions with reduced ventilation due to fibrosis or to a decrease in volume change between inspiration and expiration, each voxel of the ventilation map was sorted into three classes based on ΔSVg and SVg values: fibrosis, low ventilation and normal ventilation. Fibrosis (F, *red* in Fig. 1): lung regions with inspiratory SVg lower than α_I_ and expiratory SVg lower than α_E_. Low ventilation (LV, *yellow*): lung regions characterized by ΔSVg < β and inspiratory and expiratory SVg higher than α_I_ and α_E,_ respectively. Normal ventilation (NV, *green*): lung regions characterized by ΔSVg >  = β and inspiratory and expiratory SVg values higher than α_I_ and α_E,_ respectively.

For classification purposes, we defined “normal” thresholds based on the distribution of SVg and ΔSVg in saline-treated control animals: α_I_ and α_E_ as the 5th percentile of the inspiratory and expiratory SVg distribution, respectively, and β as the 25th percentile of the ΔSVg distribution in the saline group.

### Longitudinal imaging biomarkers

Density-based measurements. The CT attenuation histogram of lung parenchyma, derived from inspiratory images, was plotted and median, 75th percentile and interquartile range (75–25th percentile, IQR) were calculated. The 75th percentile was used to quantify the histogram tail distribution on the right-side, which is indicative of processes that increase lung density such as ground glass opacities, fibrosis and consolidations. This parameter may thus represent a useful indicator of parenchymal fibrotic disease. The interquartile range was calculated as index of heterogeneity. Each metric was calculated over the whole lung and over the upper and lower regions, as a means to disclose regional mechanisms of disease and antifibrotic drug action. Upper and lower lung regions were obtained by dividing the lungs into two regions of equal vertical size.

Ventilation-based measurements. Ventilation of lung parenchyma was plotted as a histogram and median, 75th percentile and interquartile range of ΔSVg were calculated as indexes of ventilation distribution. Unlike CT attenuation, the 75th percentile of ventilation, which quantifies the histogram tail distribution on the right-side, corresponds to normal values and decreases in case of impaired ventilation. The amount of fibrosis, low ventilated and normally ventilated regions, were quantified as the percent volume of each class. Each measurement was performed over the whole lung as well as on the upper and lower regions. To allow comparison with histology data, ΔSVg and the corresponding functional classes were also measured in the left lung and in lung thirds of equal vertical size, namely ventral, intermediate, and dorsal regions.

### Histology

Histological analysis was performed in 2 saline-, 3 BLM- and 6 Nintedanib-treated animals. At day 21, mice were sacrificed, and lungs were removed, inflated with 0.6 ml of 10% neutral-buffered formalin and fixed for 24 h. For histological assessment, the whole lungs were dehydrated in graded ethanol series, clarified in xylene and paraffin embedded. The lungs were sectioned (5 μm) with a rotary microtome (Reichert-Jung 1150/Autocut), following the dorsal plane. For analysis the whole-slide images were acquired by the NanoZoomer S-60 Digital slide scanner (Hamamatsu). Fibrotic lung injury was assessed by quantitative parameters.

### Collagen content

For each slide, ROIs were selected by dividing the left lung in three regions of equal vertical extent. To standardize image contrast, brightness and colour threshold settings, the image analysis was performed to detect areas of green-stained collagen within each ROI. The collagen content is expressed as the percentage of collagen deposition area (μm2) referred to the lung total area (μm2) within the ROI. Bronchi and blood vessels have been removed from the ROI area.

### Alveolar air area

The air area within the same ROIs was detected using a white threshold. The Air Area is expressed as the percentage of area (μm2) occupied by air referred to the lung total area (μm2) within the ROI. Bronchi and blood vessels have been removed from the ROI area.

## Statistical analysis

Statistical analysis was performed using SigmaStat version 11.0 (Systat Software) and MATLAB (The MathWorks Inc.). One-way analysis of variance was applied to compare imaging biomarkers between groups (saline, BMT and Nintedanib-treated animals). In cases in which the equal variance test and/or the normality test failed, the nonparametric Kruskal–Wallis analysis of variance on ranks was applied. Where differences were identified, a Dunnett’s or Dunn’s multiple comparison post hoc test was performed. The median value of ΔSVg and the percentages of the functional classes were compared to histomorphometric measures using a Spearman rank‐order correlation.

Values in the main body and tables are reported as medians (25–75th percentiles), and significance was determined by using a difference with *p* < 0.05.

## Supplementary Information

Supplementary Information.

## Data Availability

All datasets generated for this study are included in the article/Supplementary Information.
