# Supplementary material for: Micro-CT-derived ventilation biomarkers for the longitudinal assessment of pathology and response to therapy in a mouse model of lung fibrosis
Source: Sci Rep. 2023 Mar 17;13:4462. doi: 10.1038/s41598-023-30402-8 (PMC10023700; doi:10.1038/s41598-023-30402-8)
Supplement: Supplementary file 1 — Supplementary Information. [file 41598_2023_30402_MOESM1_ESM.docx]

**TABLES**

**Supplemental Table 1.** Histological and micro-CT derived analysis in apical, medial and caudal regions of the left lung.

|  | **Saline** | **BLM** | **BLM+NTD** |
| --- | --- | --- | --- |
| **Histology** |  |  |  |
| Collagen Area (%) |  |  |  |
| *Apical* | 3.9±1.3 | 8.3±2.7 | 7.7±3.7 |
| *Medial* | 3.5±1.2 | 8.8±3.0 | 7.1±3.1 |
| *Caudal* | 3.7±1.0 | 6.8±3.0 | 5.7±2.7 |
| Air area (%) |  |  |  |
| *Apical* | 79.0±2.1 | 58.0±14.3 | 64.4±11.3 |
| *Medial* | 77.3±3.0 | 59.5±10.8 | 60.0±6.3 |
| *Caudal* | 80.1±3.6 | 67.9±7.4 | 65.2±5.4 |
| **Micro-CT analysis** |  |  |  |
| DSVg (ml/g) |  |  |  |
| *Apical* | 0.43±0.15 | 0.05±0.06 | 0.16±0.14 |
| *Medial* | 0.37±0.11 | 0.18±0.16 | 0.32±0.15 |
| *Caudal* | 0.34±0.09 | 0.39±0.06 | 0.40±0.18 |
| Fibrosis (%) |  |  |  |
| *Apical* | 3.8±2.6 | 57.8±18.2 | 24.4±20.0 |
| *Medial* | 2.3±1.1 | 32.2±17.5 | 6.4±4.7 |
| *Caudal* | 8.4±2.3 | 2.9±2.0 | 7.0±4.4 |
| Low ventilation (%) |  |  |  |
| *Apical* | 14.3±4.7 | 12.9±2.7 | 20.4±6.5 |
| *Medial* | 14.1±3.9 | 13.0±4.0 | 17.9±5.7 |
| *Caudal* | 11.6±0.1 | 12.2±5.4 | 13.1±4.5 |
| Normal ventilation (%) |  |  |  |
| *Apical* | 79.6±8.1 | 20.1±19.3 | 49.4±19.9 |
| *Medial* | 80.8±5.9 | 50.6±20.9 | 71.7±10.4 |
| *Caudal* | 74.3±4.7 | 82.3±7.2 | 75.6±8.4 |
